# Is There a Place for Dietary Fiber Supplements in Weight Management?

**DOI:** 10.1007/s13679-012-0016-9

**Published:** 2012-04-13

**Authors:** Michael R. Lyon, Veronica Kacinik

**Affiliations:** 1 Canadian Center for Functional Medicine, 1550 United Boulevard, Coquitlam, BC Canada V3K 6Y2; 2 Food, Nutrition and Health Program, University of British Columbia, 1550 United Boulevard, Coquitlam, BC Canada V3K 6Y2

**Keywords:** Dietary fiber, Obesity, Appetite regulation, Glycemic index, GLP-1, PYY, CCK, Oxyntomodulin, L-cells, Free fatty acid receptors, Bile acid receptors

## Abstract

Inadequate dietary fiber intake is common in modern diets, especially in children. Epidemiological and experimental evidence point to a significant association between a lack of fiber intake and ischemic heart disease, stroke atherosclerosis, type 2 diabetes, overweight and obesity, insulin resistance, hypertension, dyslipidemia, as well as gastrointestinal disorders such as diverticulosis, irritable bowel disease, colon cancer, and cholelithiasis. The physiological effects of fiber relate to the physical properties of volume, viscosity, and water-holding capacity that the fiber imparts to food leading to important influences over the energy density of food. Beyond these physical properties, fiber directly impacts a complex array of microbiological, biochemical, and neurohormonal effects directly through modification of the kinetics of digestion and through its metabolism into constituents such as short chain fatty acids, which are both energy substrates and important enteroendocrine ligands. Of particular interest to clinicians is the important role dietary fiber plays in glucoregulation, appetite, and satiety. Supplementation of the diet with highly functional fibers may prove to play an important role in long-term obesity management.

## Introduction

The “eat less and exercise more” paradigm has proven to be of little value in the clinical management of the obese patient. Likewise, treatments that rely upon complex or rigorous dietary plans tend to result in poor long-term benefits with patients typically drifting back to their old habits once the novelty of the diet has worn off. Clinicians are increasingly assisting obese patients in the establishment of more achievable long goals, including the avoidance of further weight gain as a realistic end point in some patients. Rather than complex dietary regimens, most patients respond more favorably to incorporating eating strategies that help them achieve and maintain a sense of satiety while reducing their caloric intake. Significantly increasing the consumption of dietary fiber to reduce the caloric density of food and reduce the glycemic impact of the food is generally considered to play an important, if not essential, part of long-term weight management [1].

## What Is Dietary Fiber?

Dietary fiber is a non-starch polysaccharide in (mostly) plant food that is poorly digested by humans. Based on a recent US government consensus report, fiber can exist as dietary fiber (naturally occurring in food), or functional fiber (added during the processing or preparation of food or consumed separately as a supplement) [2]. Fiber can be insoluble or soluble in water. Insoluble fibers include cellulose, hemicellulose, and lignins, whereas soluble fibers include various gums, pectins, β-glucans, oligosaccharides, resistant dextrans, and resistant starches. Chitin and chitosan are indigestible amino-polysaccharides that are found in or are derived from the exoskeletons of arthropods such as crabs and lobster, as well as the cell walls of most fungi, and could functionally be regarded as fiber, although they are not recognized as fiber by most regulatory authorities. The distinction between soluble and insoluble fibers is due to the chemical properties of the fiber, resulting in its tendency to absorb water. Various physicochemical properties of fiber (viscosity, water-holding capacity, cation exchange capacity, adsorption of organic materials, and fermentability) are now thought to be fundamental to its beneficial physiologic effects. The Institute of Medicine has proposed a new definition of dietary fiber that encompasses both its physical characteristics and its physiologic effects in humans [3]. A fiber’s viscosity, its water-holding capacity, and its fermentability are the chief determinates of fiber’s physiologic effects.

The regulatory classification of fiber varies considerably in different countries. The Codex Alimentarius Commission of the World Health Organization has defined dietary fiber upon analytic methods rather than its physical or physiologic characteristics [4].

## Sources

The typical Western diet is generally lacking in sufficient dietary fiber, being composed principally of refined grains and other highly digestible sources of starch, sugar, various fats, and animal products. Children in particular are commonly fiber deficient, with daily intakes often under 5 g and with little soluble fiber. Likewise, many adults in Western society consume 5 to 10 g of fiber daily, as opposed to the 35 to 50 g that is considered desirable for optimal health [5]. Moreover, because most fiber in the Western diet is derived from cereal grains, the intake of viscous soluble fiber is typically inadequate.

A diet focusing on a large intake of vegetables and fruits as well as unrefined whole grains and legumes should be the foundation of a healthy lifestyle. With this “whole-foods” based diet, it is certain that dietary fiber intake will substantially increase [6]. Unfortunately, only a minority of the population, particularly children, are likely to adopt a largely whole-foods diet anytime in the near future. Because of this, efforts are underway to establish effective means to fortify the Western diet with dietary fiber through the use of functional fibers (various fibers as food additives or ingredients as well as the use of readily accepted fiber supplements).

## Health Effects

There is compelling epidemiologic and experimental data associating numerous disorders, at least in part, to a lack of dietary fiber. Ischemic heart disease, stroke atherosclerosis, type 2 diabetes, overweight and obesity, insulin resistance, hypertension, dyslipidemia, as well as gastrointestinal disorders such as diverticulosis, irritable bowel disease, colon cancer, and cholelithiasis are just a few of the many conditions that seem to be influenced by the adequacy of dietary fiber intake [7, 8].

Numerous studies have demonstrated that certain fibers decrease the glycemic response to food, promote satiety, lower serum cholesterol, promote bowel regularity, positively influence colonic microflora, provide nutritional substrates for colonic mucosal cells, improve mucosal barrier function, as well as aid in the sequestration and elimination of toxic and carcinogenic dietary and environmental compounds. These and other effects constantly interplay to increase or decrease the development of a wide range of health conditions.

Viscous dietary fibers have been correlated with moderation in blood glucose and cholesterol concentrations, prolonged gastric emptying, and slower transit time through the small intestine [9]. Among viscous fibers, fermentability is mostly associated with large bowel function. Rapidly fermented fiber sources provide substrates for short-chain fatty acid (SCFA) production by microflora in the large bowel, whereas slowly or incompletely fermented fiber sources improve bowel health by promoting laxation, reducing colonic transit time, and increasing stool weight [10].

## Mechanisms of Action of Dietary Fiber

Dietary fiber exerts its effects through an interaction between the physical properties it imparts to foods accompanied by a complex array of microbiological, biochemical, and neurohormonal influences. Dietary fiber can have a strong influence on the palatability of food, and may require longer periods of mastication before swallowing, thus influencing ingestive behavior. In the stomach, fiber affects the volume and viscosity of food, which has a highly significant effect on satiety [11]. This “volumetric” effect on food promotes a sense of fullness and a delay in gastric emptying, which tends to naturally result in a decrease in caloric intake. Various fibers differ dramatically in their ability to impart volume and viscosity to foods, and it has been shown that simple distention of the gastric antrum by soluble, viscous fibers leads to a sensation of satiety that tends to promote cessation of eating during meal time [12]. Viscous fiber has also been shown to slow gastric emptying, thus resulting in a prolongation of the mechanical distention of the stomach [13]. As well, viscous fiber consumption results in a delayed postprandial rise of ghrelin, the principle peripheral orexigen that promotes meal initiation. This delay in the pre-meal elevation of ghrelin is thought to result from slowed absorption of glucose and amino acids and a resultant increase in the delivery of these nutrients to the jejunum and ileum [14].

## Viscosity of Fiber

Viscosity as related to dietary fiber refers to the ability of some polysaccharides to thicken or form gels when mixed with fluids resulting from physical entanglements and hydrophilic interactions among the polysaccharide constituents within the fluid or solution [15]. Gums, pectins, and β-glucans make up the majority of viscous dietary fibers. Apples, legumes, and oats are common dietary sources of viscous fibers. The viscosity that a fiber imparts to the gastric and small intestinal contents is directly correlated with the ability of the fiber to reduce postprandial glycemic response, promote satiety, decrease serum cholesterol, and decrease serum uric acid [16, 17••]. The viscosity of fiber is also thought to play an important role in the augmentation of gut mucosal protection through the stimulation of enteral mucus production and goblet cell hypertrophy and replication [18]. Additionally, those viscous fibers that are largely fermented by colonic microflora exert a wide array of physiologic effects through the production of SCFAs (the principle energy substrates of colonocytes), the promotion of beneficial colonic microbial populations, and the augmentation of important gut-derived peptide hormones.

The viscosity of fiber is best measured by methods that quantify a hydrated fiber’s internal friction and its ability to resist flow. Viscosity is usually expressed in units of millipascal seconds or centipoise [19]. Other factors, such as shear stress (e.g., mastication, peristalsis), acid pH, dilution, and chemical components of food determine the real viscosity that a fiber will impart to food rather than just its in vitro viscosity.

The concept of the glycemic index (GI) came about through the work of Jenkins et al. [20] as they examined the impact of viscous fiber ingestion on glucose tolerance. It is now generally accepted that the GI of a carbohydrate-containing food is directly correlated to the viscosity of that food after ingestion. Viscous fibers increase small intestinal transit time, thus decreasing the speed of macronutrient digestion and absorption [14]. Along with changes in absorptive rate, prolonging the exposure of the enteral mucosa to macronutrients augments the liberation of anorexigenic peptides. Fat and protein in the proximal small bowel stimulate the release of cholecystokinin (CCK), which promotes acute postprandial satiety. As well, the elevated delivery of carbohydrates to the distal small intestine, stimulate the release of the anorexigenic peptides glucagon-like peptide 1 (GLP-1) and peptide YY (PYY) in a manner perhaps akin to that which occurs (on a greater scale) from the malabsorption accompanying gastric bypass surgery [21, 22•]. These mechanisms tend to promote satiety between meals and result in a delay in the onset of hunger.

## Water-Holding Capacity and Energy Density

The ability of a fiber to absorb and hold on to water as it transits the gut is key factor that contributes to its functional effects. Soluble fibers have the capacity to create a stable gel that results in stomach volume being occupied and a sense of satiety created without the addition of significant calories. This concept has been referred to as “caloric displacement,” meaning that the consumption of low-calorie-density food or supplemental fiber can result in the achievement of satiety with a caloric intake that is less than if the food consumed was higher in caloric or energy density.

**Fig. 1:**
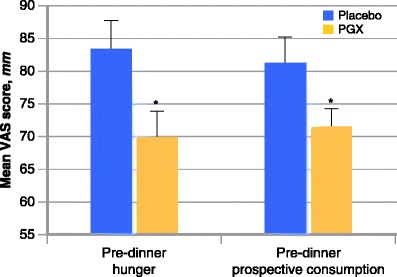
Comparison of pre-dinner mean hunger and prospective consumption scores of day 3 of the 1000-calorie diet supplement with 5 g of PGX or placebo at each meal. Values are mean±SE (*n* = 35). *Asterisk* (*) indicates significantly lower scores with PGX than the placebo supplement (*P* < 0.05). VAS—visual analogue scale

A food’s energy density consists of the net quantity of calories in a particular weight of food (usually expressed as kcal/g). Several strategies have been employed involving the addition of substantial amounts of low-energy-density foods to a meal plan. For instance, consuming a high-fiber, low-energy-density soup as a “preload” before the rest of a meal significantly reduces the ad libitum food intake for that meal while increasing feelings of satiety [23]. Similarly, consuming a large, low-energy-density salad at the onset or during a meal reduces the total ad libitum consumption of food for that meal [24••]. Recently, it has been demonstrated that a variety of high-fiber vegetables can be added to acceptable foods resulting in a significant decrease in the calorie density of the food without a negative impact upon the palatability of the food, even with children [25••]. Consuming functional fiber supplements prior to or with meals can also reduce the caloric density of meals and promote satiety. As an example, our research group recently studied the effect of adding a highly viscous functional fiber supplement (PGX®) to each meal with food, or in liquid meal replacements in women on a low-calorie diet. In this double-blind placebo-controlled trial, each 5-g dose of the highly viscous fiber was estimated to hold approximately 1 L of water in its passage through the stomach and small intestine, essentially adding 3 L or over 6 lb of non-caloric food mass to the daily food intake. In this study we were able to show that the viscous fiber supplement significantly reduced hunger feelings and promoted satiety during a period of significant caloric reduction (Fig. 1) [26••].

Those who work in the field of obesity management generally accept the necessity of teaching patients practical strategies that promote the consumption of low-calorie-density, highly volumetric foods in the dietary management of overweight and obesity [27••, 28, 29]. Functional fiber supplements may play a significant role in assisting patients in the achievement of consistent reductions in the energy density of their diets, especially during periods of significant caloric reduction.

## Prebiotic Effects of Fiber

It is increasingly recognized that certain forms of fiber are fermentable, providing important metabolic substrates in the metabolism of gut flora. By definition, humans lack the enzymes specific to digest fiber. In the case of insoluble fiber, most gut microbes cannot utilize this as an energy source, and so it is typically excreted without any molecular alteration other than by forming a surface for the adsorption of water, organic matter, and cations. Soluble fibers vary in their fermentability and in the specific microbes that can utilize them as substrates. The term “prebiotic” was coined by Gibson and Roberfroid [30, 31] in 1995, and was defined as an indigestible carbohydrate that is fermentable in the lower gastrointestinal tract that selectively promotes the growth of desirable (prebiotic) microflora and is associated with a positive health outcome. Although there may be exceptions, prebiotics tend to reduce populations of potentially pathogenic flora while promoting desirable commensals such as *Bifidobacteria*.

Recent evidence suggested that this effect might play an important role in both the reduction in adiposity as well as a decline in the contribution of adipocytes to inflammation. It has been demonstrated in animal models that prebiotic supplementation might reduce the development of large adipocytes (those that predominate in the visceral compartment), which are highly involved in the overactivation of a wide variety of inflammatory processes associated with overeating, obesity, diabetes, cardiovascular disease, and pain [32, 33].

Pathways of inflammation associated with atopic disease may also be intimately associated with gut flora. Both probiotics and prebiotics are believed to hold significant promise in the prevention and treatment of allergies and atopic disorders, such as eczema [34–37], and they may have the potential to play a significant role in the management of inflammation as it relates to metabolic syndrome and other obesity-related comorbidities such as nonalcoholic steatohepatosis [38, 39].

Lack of sufficient intake of prebiotics early in life may have lasting ill effects on glucoregulation, which may result in a substantial predilection toward obesity, diabetes, and cardiovascular disease later in life [40]. Unfortunately, there is little effort being made to increase fermentable soluble fiber intake in infants and children. This may have serious consequences and may be one of the many reasons for the current obesity epidemic. The food industry has just begun to respond to this with the introduction of prebiotic-fortified infant formulas. Because breast milk contains significant quantities of prebiotic oligosaccharides, adding analogous agents to infant formulas would help these formulas to more closely mimic breast milk. This strategy results in a gut flora predominated by *Bifidobacteria*, rather than potential pathogens such as *Clostridia* and *Enterobacter* that tend to predominate in formula-fed infants [41]. There is now a growing body of evidence that alterations in gut flora (eg, a relative absence of *Bifidobacteria*) are significantly associated with obesity [42]. In this regard, prebiotic fiber supplementation may play a clinically important role in the promotion of a more desirable gut microbiological milieu.

## Neuroendocrine Effects of Fiber

One of the most intriguing and rapidly unfolding discoveries is related to the role played by dietary fiber in the modulation of important neuroendocrine physiology, which may be related fundamentally to the etiology of obesity and related conditions. Of particular interest is the impact of fiber on the density and activity of a specialized enteroendocrine cell known as the L cell [43]. The L cell is located throughout the terminal ileum and colon, and it is responsible for the secretion of the peptide hormones GLP-1, PYY, and oxyntomodulin [44]. After a meal, oxyntomodulin and PYY are released synchronously and they both act as potent anorexigens. The rapid rise of these peptides signals a change in energy status to the brain and it also acts locally to enhance digestive and metabolic processes. GLP-1 is an incretin hormone that also plays a pivotal role in glucoregulation through the stimulation of accurately timed insulin secretion and suppression of inappropriate glucagon secretion from the pancreas. It has been established that diminished GLP-1 production plays a central role in the etiology of diabetes, a discovery that has led to the development of an important class of diabetes drugs, the incretin analogues. Augmentation of PYY and GLP-1 are thought to play a central role in the regulation of the “ileal brake.” The ileal brake is a feedback mechanism that results in inhibition of proximal gastrointestinal motility and secretion when nutrients and nutrient metabolites arrive in sufficient quantities to the luminal surface of the ileum. Animal and human studies show that activation of the ileal brake by local perfusion of the ileum with nutrients increases feelings of satiety and reduces ad libitum food intake while slowing gastric and proximal intestinal transit [45].

It has been recently shown that (bariatric) gastric bypass surgery frequently results in rapid amelioration of diabetes that often precedes significant weight loss [46]. Current evidence points to a rapid and sustained increase in circulating GLP-1 and PYY after this procedure that has profound effects on appetite and glucoregulation. It is now thought that the malabsorption of macronutrients and rapid gastrointestinal transit after gastric bypass results in an increased delivery of carbohydrates and their fermentation products (SCFAs) as well as bile acids, both of which activate L cells via free fatty acid (FFA) receptors and bile acid receptors [47–51].

It is most interesting that fermentable soluble fiber has the potential to generate a significant supply of SCFAs that might mimic, on a lesser scale, the mechanism of gastric bypass surgery through stimulation of FFA receptors with a resultant increase in GLP-1 and PYY and augmenting the ileal brake [52–54]. Viscous soluble fiber also effectively sequesters bile acids, reducing their usual absorption through the jejunum and delivering them to the same L cells where they stimulate bile acid receptors. Thus, viscous soluble fiber that is also fermentable may exert an appetite-reducing and glucoregulating effect through L-cell activation via both FFA receptors and bile acid receptors as well as by suppression of the orexigenic (appetite-stimulating) hormone ghrelin and augmentation of CCK secretion [55–57].

Sequestration of bile acids by viscous soluble fiber is also known to be a principle mechanism by which viscous fiber lowers serum cholesterol, because sequestration of bile acids decreases the enterohepatic recycling of bile acids, a major cholesterol reservoir for the human [58].

## Effect of Fiber on Postprandial Glycemia

Dietary recommendations for weight management usually include advice regarding the quantity of carbohydrates or the percentage of daily calories from carbohydrate-containing foods. A more meaningful recommendation might be based upon the GI (the quantitative effect of a food on postprandial glycemia) and the glycemic load (GL; the mathematical product of the GI and carbohydrate content). The GI of carbohydrate-containing foods has long been known to be directly related to the quantity and form of dietary fiber in the food [59]. Recently, it has been verified that the GI and GL are more important determinants of glycemic and insulinemic response than that of carbohydrate intake alone [60, 61]. This may have profound implications in the management of obesity and related conditions. In diabetes, continuous glucose monitoring has confirmed that GI and GL predict glycemic variability independent from total carbohydrate intake [62].

Low GI-based diets have been shown to promote satiety and reduce postprandial insulinemia [63]. However, the evidence in favor of low GI/GL is inconsistent with many weight loss studies showing only a trend in favor of this approach [64]. Long-term adherence to a low GI diet may be part of the problem in some studies. Regular use of viscous fiber supplements may help obese subjects to consistently achieve a lower GL while eating a diet that they can reasonably maintain.

Maintenance of weight after weight loss interventions is an elusive goal that has recently become the focus of several important studies. The Diogenes (Diet, Obesity, and Genes) study was designed to assess the efficacy of moderated fat diets that vary in protein content and GI in the prevention of weight regain and obesity-related risk factors after weight loss [65••]. In this trial, the group maintained on the low GI diet with moderately higher protein (25% of calories from protein) had the highest compliance, the lowest dropout rate, and was the only group that did not regain weight by the end of the 26-week weight maintenance intervention period. Data from the same study concluded that the low GI diets also had a more substantial effect on the reduction of inflammation (as evidenced by C-reactive protein) than the other diet interventions [66]. This finding is in keeping with other studies pointing to a substantial benefit of dietary fiber in the reduction of CRP, oxidative stress, and proinflammatory cytokines [67–69].

The consistent addition of functional fiber supplements to the diet may present a practical means to achieve meaningful reductions in the GI/GL. Recently, our research group was involved in several studies looking at the effects of adding a novel, highly viscous functional fiber supplement (PGX®) to various foods on GI, serum cholesterol [71], hunger, and satiety in healthy humans [26••, 71••, 72, 73••, 74, 75]. The novelty of this fiber relates to its viscosity, which is higher than other fibers thus studied and the fact that its viscosity slowly evolves once hydrated. This allows consumption before palatability is significantly affected. This viscous fiber is fermentable and prebiotic [76] and it is tasteless, and disperses readily when added to food or mixed with beverages. Its viscosity develops several minutes after initial hydration, making it easy to consume the small amounts needed to create a highly viscous and volumetric gastric milieu, leading to its resultant physiologic effects.

In several studies, we have shown that the GI of food can be substantially reduced with a small preload or intra-meal load of this highly viscous fiber [71••, 72, 73••, 75]. It has also been shown to promote satiety and reduce subsequent food consumption [25••]. In a human clinical trial, this novel viscous fiber has been associated with an increase in the appetite-reducing hormone PYY [77]. In studies of Zucker diabetic fatty rats, the effects of diets supplemented with this highly viscous fiber were compared with other dietary fibers. Only the diet supplemented with the highly viscous fiber substantially decreased postprandial blood glucose and insulin secretion, decreased hepatic fatty infiltration, and preserved pancreatic β-cell mass [78]. These effects were accompanied by an increase in the production of the glucoregulatory incretin hormone GLP-1 [79].

## Recommended Intake of Fiber for Weight Management

The dietary fiber intake for typical Americans is usually less than desirable, with typical intakes averaging only 14 to 15 g/day and children consuming less than 5 g/day [4]. The American Dietetic Association currently recommends that healthy adults should consume 20 to 35 g of fiber per day and children should consume at least 5 g/day plus 1 g for every year of their age [80]. They point out that insoluble, nonfermentable, and low-viscosity fiber is principally consumed to promote laxation and other aspects of colon health, whereas viscous soluble fibers are necessary to reduce serum cholesterol, blunt postprandial glycemic response, and promote satiety. A fiber-rich meal, particularly a meal high in viscous, soluble fiber, is processed more slowly, promoting earlier satiety, and is frequently less calorically dense and lower in fat and added sugars. All of these characteristics are typical of a dietary profile optimized to treat and prevent obesity. In one study, consisting of 252 middle-aged women it was observed that over a 20-month period participants lost an average of 4.4 lb in association with an 8-g increase in dietary fiber per 1000 kcal [81]. Likewise, in a prospective cohort study of nearly 30,000 men, a dose–response relationship was found between fiber intake and weight gain over a period of 8 years. They reported that for every 40-g/d increase in whole-grain intake, weight gain decreased by 1.1 lb. Moreover, the addition of supplemental bran seemed to play an important role in the reduction of weight gain by 0.8 lb per 20 g/d intake [82]. Pal et al. [83••] at Curtin University in Australia demonstrated the utility of high-dose psyllium given as a premeal supplement (12 g before each of 3 meals) as an adjunct to a short-term, calorie-reduced weight loss program. Other studies have demonstrated conflicting results from supplemental fiber in short-term weight management [71, 85].

## Conclusions

Most clinicians in the field of obesity medicine agree that dietary intervention for long-term weight management should include practical eating strategies that promote and maintain satiety to improve compliance and to minimize discomfort in patients working to reduce energy intake. In this regard, many clinicians now recommend volumetric, low-calorie-density, low GL diets supplemented with moderate amounts of protein. Increasing the intake of fiber by consuming more high-fiber foods should play a central role in this regard. These changes alone may not be sufficient to bring about long-term weight reduction [85]. Supplementation of the diet with functional fiber supplements may significantly augment high-fiber eating strategies by further promoting satiety and reducing cardiometabolic risk factors. Long-term clinical trials looking at the optimal form, quantity, and frequency of dietary fiber supplements are greatly needed to clarify their potential in the long-term management of obesity.
